# Supplementary material for: Efficacy of two rounds of albendazole treatment on soil-transmitted helminths in schoolchildren, Yunnan Province, China
Source: Nat Commun. 2026 Jan 8;17:292. doi: 10.1038/s41467-025-64883-0 (PMC12783811; doi:10.1038/s41467-025-64883-0)
Supplement: Supplementary file 2 — Reporting Summary [file 41467_2025_64883_MOESM2_ESM.pdf]

nature portfolio

Corresponding author(s): Prof Darren Gray

Last updated by author(s): Jan 16, 2025

Reporting Summary

Nature Portfolio wishes to improve the reproducibility of the work that we publish. This form provides structure for consistency and transparency in reporting. For further information on Nature Portfolio policies, see our Editorial Policies and the Editorial Policy Checklist.

Statistics

For all statistical analyses, confirm that the following items are present in the figure legend, table legend, main text, or Methods section.

n/a	Confirmed	
☐	☒	The exact sample size (*n*) for each experimental group/condition, given as a discrete number and unit of measurement
☐	☒	A statement on whether measurements were taken from distinct samples or whether the same sample was measured repeatedly
☐	☒	The statistical test(s) used AND whether they are one- or two-sided<br>*Only common tests should be described solely by name; describe more complex techniques in the Methods section.*
☐	☒	A description of all covariates tested
☐	☒	A description of any assumptions or corrections, such as tests of normality and adjustment for multiple comparisons
☒	☐	A full description of the statistical parameters including central tendency (e.g. means) or other basic estimates (e.g. regression coefficient) AND variation (e.g. standard deviation) or associated estimates of uncertainty (e.g. confidence intervals)
☐	☒	For null hypothesis testing, the test statistic (e.g. *F*, *t*, *r*) with confidence intervals, effect sizes, degrees of freedom and *P* value noted<br>*Give P values as exact values whenever suitable.*
☒	☐	For Bayesian analysis, information on the choice of priors and Markov chain Monte Carlo settings
☒	☐	For hierarchical and complex designs, identification of the appropriate level for tests and full reporting of outcomes
☐	☒	Estimates of effect sizes (e.g. Cohen's *d*, Pearson's *r*), indicating how they were calculated

Our web collection on statistics for biologists contains articles on many of the points above.

Software and code

Policy information about availability of computer code

Data collection	Data on demographic and parasitological surveys were collected using questionnaires and laboratory forms, respectively. Data were entered by 2 different data encoders in a customized password-protected data entry system developed using Microsoft Access.
Data analysis	Software used for analysis: SAS (r) Proprietary Software 9.4 (TS1M7) [Copyright (c) 2016 by SAS Institute Inc., Cary, NC, USA, Licensed to Queensland Institute of Medical Research (QIMR)- Genetics and Population Health, Site 10008492.

For manuscripts utilizing custom algorithms or software that are central to the research but not yet described in published literature, software must be made available to editors and reviewers. We strongly encourage code deposition in a community repository (e.g. GitHub). See the Nature Portfolio guidelines for submitting code & software for further information.

Data

Policy information about availability of data

All manuscripts must include a data availability statement. This statement should provide the following information, where applicable:

- Accession codes, unique identifiers, or web links for publicly available datasets
- A description of any restrictions on data availability
- For clinical datasets or third party data, please ensure that the statement adheres to our policy

Data availability:
Data supporting the finding of this study are not publicly available due to participant confidentiality. However, the data may be available from the corresponding author upon reasonable request

Code availability:
Custom code for data processing and analysis will be available from the corresponding author upon reasonable request. This is to ensure the codes are used appropriate use to maintain the integrity of the research through direct communication with interested parties.

Research involving human participants, their data, or biological material

Policy information about studies with human participants or human data. See also policy information about sex, gender (identity/presentation), and sexual orientation and race, ethnicity and racism.

Reporting on sex and gender	Participants of any sex were eligible for inclusion for this study, from which specimens and data for the present study are derived. The study recruited roughly the same numbers of male and females (46.9% vs 50.7%). We did not perform any analysis stratified by sex.
Reporting on race, ethnicity, or other socially relevant groupings	The current study conducted in Bulang Shan region located in Menghai county, Yunnan Province, China and therefore the study participants are primarily Chinese schoolchildren. The participants were recruited from 22 schools around the region without consideration of ethnicity or targeting any key populations.
Population characteristics	For demographic characteristics of the study population, please refer to Table 1.<br><br>We acknowledge that this study lacks a comparison group as a limitation which means no direct measure for comparative effectiveness. The current study was conducted to assess the added benefit of a second of 400mg dose of albendazole in addition to the standard regimen (single round 400mg dose) in the context of a public health deworming program in a highly endemic setting, thus assignment of a control group (placebo) was unattainable and unethical to implement.
Recruitment	Before commencement of the study, informed consent was obtained from the parents or legal guardians of the study participants. For children aged >12 years, both informed consent from the parent or guardian and assent from the study participant were obtained.
Ethics oversight	The study was approved by the Yunnan Institute of Parasitic Diseases, (China), Queensland Institute of Medical Research Human Research Ethics Committee (No. P1271), and the Australian National University Human Ethics Committee (No. 2014/356).

Note that full information on the approval of the study protocol must also be provided in the manuscript.

Field-specific reporting

Please select the one below that is the best fit for your research. If you are not sure, read the appropriate sections before making your selection.

[x] Life sciences [ ] Behavioural & social sciences [ ] Ecological, evolutionary & environmental sciences

For a reference copy of the document with all sections, see nature.com/documents/nr-reporting-summary-flat.pdf

Life sciences study design

All studies must disclose on these points even when the disclosure is negative.

Sample size	The sample size was estimated based on treatment efficacy of single dose albendazole (60%) and repeated dose albendazole (85%) on STH infections, α=0.05 and , 1-β=0.90, a sample size of 128 STH positive children was required. Considering some children may miss the second treatment (20% attrition), thus a total of 153 STH positive children was targeted at the beginning of the study.<br><br>To ensure the sample size can be achieved, the present study recruited 453 schoolchildren and screened for STH infection. Of the these, 375 were found STH positive and enrolled for follow-up. This number exceeded our sample size calculation of 153 schoolchildren.
Data exclusions	Children negative for all STH species infections at baseline were excluded.

Replication	The study implemented a standardized data collection procedure to ensure consistent procedures were followed across all participants.<br><br>The stool samples were analyzed by Kato-katz technique. For quality control, 10% of the slides were rechecked by independent microscopists unaware of the initial results.
Randomization	The study participants were not randomized. All individuals underwent the same treatment regimen.
Blinding	For this study, stool samples at baseline and follow-up were read by the laboratory staff/microscopists who were blinded by the study outcomes.

Reporting for specific materials, systems and methods

We require information from authors about some types of materials, experimental systems and methods used in many studies. Here, indicate whether each material, system or method listed is relevant to your study. If you are not sure if a list item applies to your research, read the appropriate section before selecting a response.

Materials & experimental systems

n/a	Involved in the study
☒	☐ Antibodies
☒	☐ Eukaryotic cell lines
☒	☐ Palaeontology and archaeology
☒	☐ Animals and other organisms
☒	☐ Clinical data
☒	☐ Dual use research of concern
☒	☐ Plants

Methods

n/a	Involved in the study
☒	☐ ChIP-seq
☒	☐ Flow cytometry
☒	☐ MRI-based neuroimaging

Plants

Seed stocks	Not applicable
Novel plant genotypes	Not applicable
Authentication	Not applicable
